# *BSxCuBE-Web* – a web application for bioSAXS high-throughput collection and experimental control

**DOI:** 10.1107/S1600577526003528

**Published:** 2026-04-21

**Authors:** Jean Baptiste Florial, Marcus Oscarsson, Antonia Beteva, Stuart Fisher, Jérôme Kieffer, Antonino Calio, Hayden Fisher, Martha Brennich, Dihia Moussaoui, Anton Popov, Montserrat Soler-Lopez, Petra Pernot, Mark D. Tully, Andrew A. McCarthy

**Affiliations:** a European Molecular Biology Laboratory (EMBL) 71 avenue des Martyrs, CS 9018 38042 Grenoble France; b Molecular Crystallography, Imaging and Scattering Group (MXIS) European Synchrotron Radiation Facility (ESRF) 71 avenue des Martyrs F-38000Grenoble France; Australian Synchrotron, Australia

**Keywords:** web technologies, biological small-angle X-ray scattering, experimental control, automation

## Abstract

The biological small-angle X-ray scattering (bioSAXS) beamline BM29 at the ESRF, operated by the ESRF–EMBL Joint Structural Biology and bioImaging Group (JSBIG), resumed user operation in September 2020 following the ESRF extremely brilliant source (EBS) upgrade. To exploit the high quality of X-ray beam delivered by this new fourth-generation synchrotron source, BM29 underwent significant refurbishment, including source optimization, instrument upgrades, and a complete redesign of the whole experimental control system. Here, we introduce the *BioSAXS Customized Beamline Environment* (*BSxCuBE-Web*), a new open-source, web-based platform designed to streamline, automate and enhance bioSAXS data collection. *BSxCuBE-Web* offers an intuitive and user-friendly interface for expert and non-expert users alike, to easily define and run bioSAXS experiments, as well as to monitor both raw and processed data. Its adaptable architecture facilitates its deployment on beamlines beyond the ESRF. In 2025 alone, *BSxCuBE-Web* supported more than 97 experiments involving over 256 users at BM29. The interface has been successfully validated using a wide range of biological samples and continues to be actively developed in response to bioSAXS user feedback and evolving experimental requirements. User experience reports have been overwhelmingly positive, highlighting significant improvements in efficiency, accessibility, and reliability during data collection.

## Abstract 2

A new open-source web-based graphical user interface, *BioSAXS Customized Beamline Environment* (*BSxCuBE-Web*), for the experimental control of bioSAXS experiments on beamline BM29 at the ESRF–EBS is presented.

## 1. Introduction

The ESRF–EMBL Joint Structural Biology and bioImaging Group (JSBIG) operates a modern suite of synchrotron-based beamlines, together with other facilities on the Grenoble EPN campus that provide integrated structural biology services (McCarthy *et al.*, 2025 ▸). BM29 is a dedicated biological small angle X-ray scattering (bioSAXS) beamline for the study of biological macromolecules in solution, operational since 2012. The beamline originally began as an endstation on the undulator source ID14-3 before being significantly improved and relocated to a dedicated bending magnet source on BM29 in 2012 (Pernot *et al.*, 2013 ▸) as part of the ESRF Phase-1 upgrade program (Mueller-Dieckmann *et al.*, 2015 ▸). This experimental setup included a temperature-controlled robotic liquid-handling sample changer (SC) with an integrated sample exposure unit (SEU) (Round *et al.*, 2015 ▸) and a high-performance liquid chromatography (HPLC) system for size exclusion chromatography (SEC)-SAXS measurements (Round *et al.*, 2013 ▸). A dedicated and user-friendly graphical interface, the *BioSAXS Customized Beamline Environment* (*BSxCuBE*) was developed for BM29 (Pernot *et al.*, 2013 ▸) and used until shutdown for the ESRF–Extremely Brilliant Source (EBS) upgrade in 2019. The original version of *BSxCuBE* was built on the ESRF ‘framework4’, a library with model-view-controller (MVC) architecture that uses bricks instead of widgets. It was built with Python and *Qt4*.

BM29 underwent extensive refurbishment during the ESRF–EBS upgrade (Raimondi *et al.*, 2023 ▸) to maximize its scientific potential following completion of this new fourth generation synchrotron source (Tully *et al.*, 2023 ▸). This included an in-vacuum PILATUS 2M detector (DECTRIS, Baden, Switzerland), a new sample changer (ARINAX, Moirans, France), an SEU for standard capillary measurements (SEU-2A), and a flexible SEU for specialized sample delivery devices such as microfluidic chips (SEU-2B). The ESRF–EBS shutdown period also provided the opportunity to completely refactor the whole software stack on BM29 using a modern open-source suite of control software. This included installation of the ESRF-developed *BeamLine Instrument Support Software* (*BLISS)* (Guijarro *et al.*, 2023 ▸) and *FreeSAS*, a new open-source data analysis software to reduce and analyse SAXS data (Kieffer *et al.*, 2022 ▸), an evolution of the previous version developed using the *EDNA* framework (Brennich *et al.*, 2016 ▸).

Building on the successful deployment of the web-based experimental interface *MXCuBE-Web* from the *MXCuBE* collaborative project (Oscarsson *et al.*, 2019 ▸) on the macromolecular crystallography (MX) JSBIG beamlines, we adopted a similar strategy and developed a web-based version of *BSxCuBE* for the refurbished BM29. The primary objective was to develop an intuitive and user-friendly interface for bioSAXS experimental setups on BM29. *BSxCuBE-Web* controls the sample changer, HPLC, and coordinates experimental protocols. After data collection, it launches automated data analysis routines uploaded to the experimental database dedicated to bioSAXS experiments, ISPyB for bioSAXS (De Maria Antolinos *et al.*, 2015 ▸), and to the new ESRF data portal, DRAC (De Maria *et al.*, 2024 ▸). *BSxCuBE-Web* represents a significant improvement over *BSxCuBE*, providing more data collection options and hardware control interfaces than the previous version. Here, we describe the technology used and how *BSxCuBE-Web* is currently deployed on BM29.

## 2. The evolving state of beamline control software for bioSAXS beamlines

The development of *BSxCUBE-Web* has been guided by software strategies implemented across synchrotron facilities worldwide. Many of these facilities utilize distinct software design philosophies to address the challenges of modern bioSAXS experiments, such as high-throughput screening, SEC-SAXS, and time-resolved experiments. In many cases, the growing complexity of modern experiments has led to a departure from generic control systems towards the development of specialized graphical user interfaces (Table 1 ▸).

One common approach is the use of facility-wide frameworks, such as the *Generic Data Acquisition* (*GDA*) system deployed at Diamond Light Source (Cowieson *et al.*, 2020 ▸) or the TANGO-based *Sardana* suite used at ALBA (https://sardana-controls.org). This approach allows centralized maintenance and a consistent user experience across beamlines. In contrast, other beamlines have opted for entirely bespoke systems, like *BECQUEREL* deployed at EMBL-Hamburg@PETRA III, which is highly tailored for the specific hardware on the P12 beamline (Hajizadeh *et al.*, 2018 ▸). A third approach involves the adaptation of established software from adjacent fields, principally MX. This is exemplified by the development of a customized version, *Blu-Ice for SAXS* (Classen *et al.*, 2010 ▸), for the SIBYLS beamline at the Advanced Light Source, which was based on *Blu-Ice* for MX experimental control at Stanford Synchrotron Radiation Source (McPhillips *et al.*, 2002 ▸) that is still under active development.

Regardless of development origin, an increasingly popular architectural approach has been to develop a modern, high-level application layer that communicates with a facility-wide standardized hardware control system. This layered approach combines centralized control and maintenance of the core functionality while allowing the flexibility to develop technique-specific applications. This trend is visible at facilities like the National Synchrotron Light Source II, which uses *Bluesky* (Allan *et al.*, 2019 ▸), a high-level Python environment that other synchrotrons such as the Advanced Photon Source and Australian Synchrotron are now adopting. While many acquisition software solutions can be adapted to a more layered structure schema, our approach was to leverage the already existing dedicated management of core controls at ESRF, allowing beamline scientists and specialist software engineers to focus their efforts on developing user-facing applications.

*BSxCuBE-Web* follows this layered philosophy. As a foundation, it uses ESRF’s facility-wide Python-based control framework *BLISS* (Guijarro *et al.*, 2023 ▸) to handle real-time hardware communication, while providing a highly optimized, user-focused web interface specifically designed to remove the complexity of modern bioSAXS experiments. This architecture allows *BSxCuBE-Web* developers to solely focus on improving the user experiment and scientific productivity, while the complexities of hardware control remain isolated within the *BLISS* layer.

## 3. Design of *BSxCuBE-Web*

The development of *BSxCuBE-Web* is the result of the close JSBIG collaboration between ESRF and EMBL Grenoble, with both institutes contributing scientific expertise and software engineering resources. This collaborative approach ensures that the platform remains tailored to meet the specific needs of the bioSAXS user community, while also being continuously optimized and expanded by a dedicated team of scientists and developers. By pooling knowledge and resources, *BSxCuBE-Web* continues to remain at the forefront of automated bioSAXS data collection and analysis, responding to the evolving needs of the European structural biology user community.

*BSxCuBE-Web* enables the automation of key steps in experimental procedures, such as sample environment control, specification of data acquisition parameters, and queuing of experimental data collections. It also integrates the SEU-2B advanced sample environment controls, enabling precise 2D spatial scanning and microfluidic pump coordination directly from a unified web browser interface. This allows users to focus on their scientific objectives rather than technical operations. The *BSxCuBE-Web* graphical user interface (GUI) offers a simple and intuitive layout that enables users to easily navigate through the interface and configure experiments, monitor data collection in real time, and quickly visualize results. This reduces the complexity of bioSAXS experiments for non-expert users who may be unfamiliar with BM29. To further help non-expert users, *BSxCuBE-Web* also provides feedback through log messages.

*BSxCuBE-Web* integrates a simple-to-use queuing system to allow researchers to schedule and run multiple bioSAXS experiments automatically, enabling efficient and high-throughput data collection. This queuing system minimizes down-time between experiments and maximizes beam time usage, particularly during longer experimental sessions or when processing a large number of samples. Users can conveniently prepare batches of samples, specify the corresponding parameters for each, and then add them to the queue through the *BSxCuBE-Web* interface. This is especially useful when working with robotic systems such as the BioSAXS Sample Changer or the HPLC system (Round *et al.*, 2015 ▸; Tully *et al.*, 2023 ▸), which can load, process and measure multiple samples sequentially without manual intervention.

*BSxCuBE-Web* facilitates the visualization of both raw and processed data immediately after collection (Kieffer *et al.*, 2022 ▸). These online feedback tools ensure that users can assess data quality in real time and adjust data collection parameters if necessary to optimize subsequent measurements. In addition to manually adjusting experimental parameters, users have the flexibility to add, remove, or reorder items in the queue while it is running. This dynamic queue management supports real-time adjustments based on immediate data feedback or changing experimental needs. For instance, if preliminary results indicate that further data are needed for a specific sample, the user can reprioritize the queue accordingly.

For more experienced users and beamline staff, *BSxCuBE-Web* provides extended configuration options concealed in the standard interface. These options, accessible via a dedicated beamline setup menu, include advanced control parameters such as selecting the X-ray energy or configuring beamline-specific options such as slit alignments. As a web-based application, *BSxCuBE-Web* supports secure remote access, enabling users to control their experiments remotely. This feature is particularly useful for beamline staff who need to troubleshoot technical issues outside regular working hours and can also be used by larger beamline allocation groups, which often need to coordinate multiple experiments and monitor automated data collections remotely during their scheduled beam time. However, a physical user presence at the beamline remains required to load samples, and especially for column equilibration and running of SEC-SAXS experiments.

By balancing usability for non-experts with advanced control options for experienced users, *BSxCuBE-Web* ensures that a wide range of researchers can conduct bioSAXS experiments effectively, improving both accessibility and overall user productivity. We anticipate that this dual accessibility design will broaden the scientific community’s engagement in the use of the BM29 beamline, leading to more diverse applications and innovative discoveries.

## 4. *BSxCuBE-Web* technology stack

**Figure 1:**
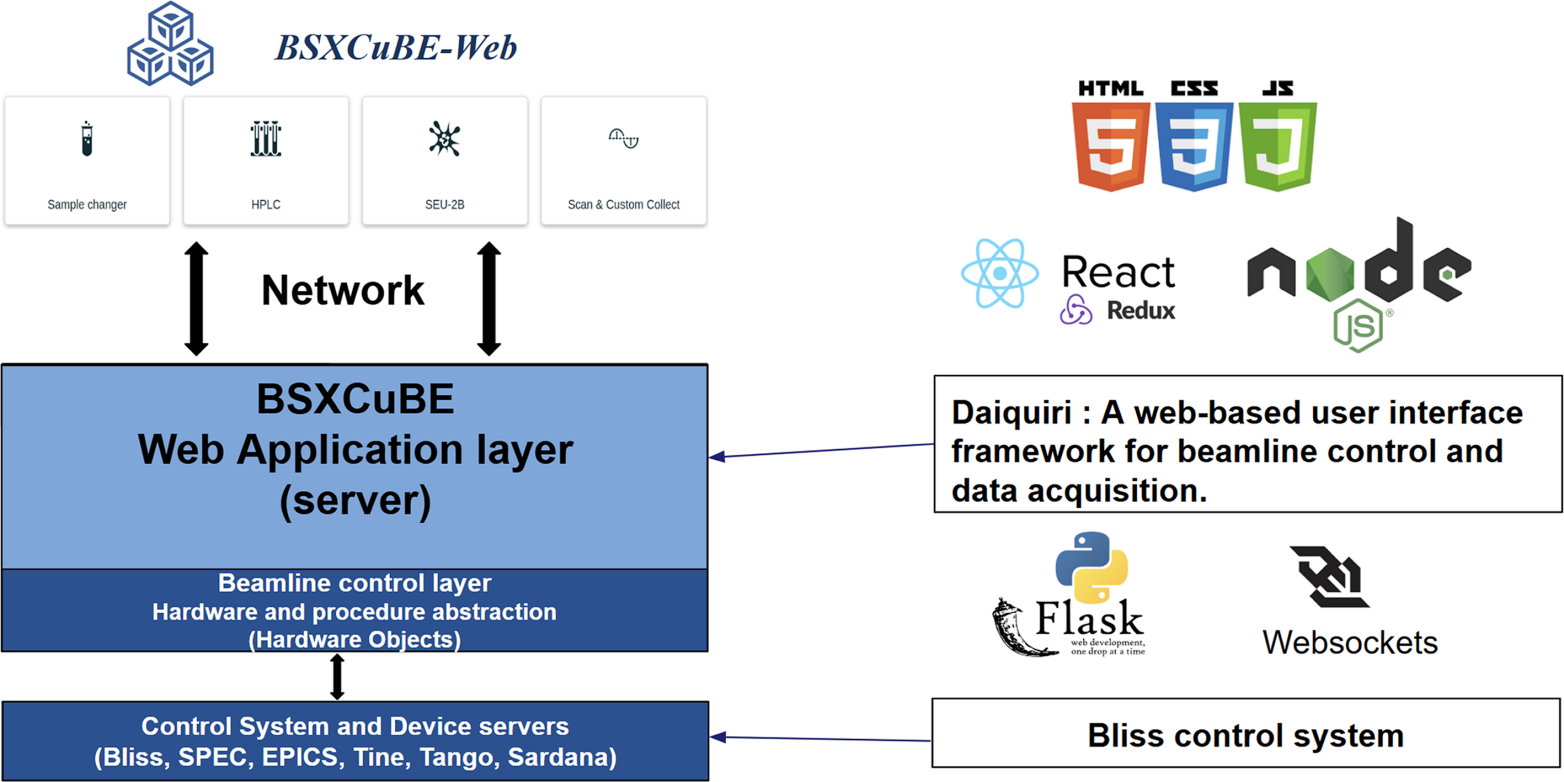
Schematic of the *BSxCuBE-Web* architecture and technology stack, showing the software components implemented at each layer.

The development of *BSxCuBE-Web* leverages a modern and powerful technology stack (Fig. 1 ▸) to create an efficient and user-friendly interface for high-throughput bioSAXS data collection and experimental control. The front-end user interface (UI) is built using the React JavaScript library together with Bootstrap 5, ensuring a responsive and consistent design that delivers a seamless user experience. Hypertext markup language (HTML) and cascading style sheets (CSS) underpin the structural and stylistic foundation for the web interface, delivering an intuitive and accessible layout. The front-end was initially implemented in JavaScript (ES2015) and later upgraded to ESNext standards. Since late 2024, new components have been progressively migrated to TypeScript. Currently, approximately 42% of front-end files (35% by line count) are written in TypeScript, following an incremental migration strategy aimed at improving type safety and maintainability. Redux is employed as the state management tool, ensuring that the UI remains synchronized with real-time updates, which is critical for effective experimental monitoring and control.

On the server side, the *Representational State Transfer* (*REST*) application programming interface (API) is implemented in Flask, a lightweight Python-based web application framework, providing the bridge between the web interface, the *BLISS* control system, and the data analysis pipelines. To maintain continuous, low-latency communication, *WebSockets* are used to deliver real-time experiment status, scan data, and results to the user without manual page refresh.

### 4.1. Back-end and control system

**Figure 2:**
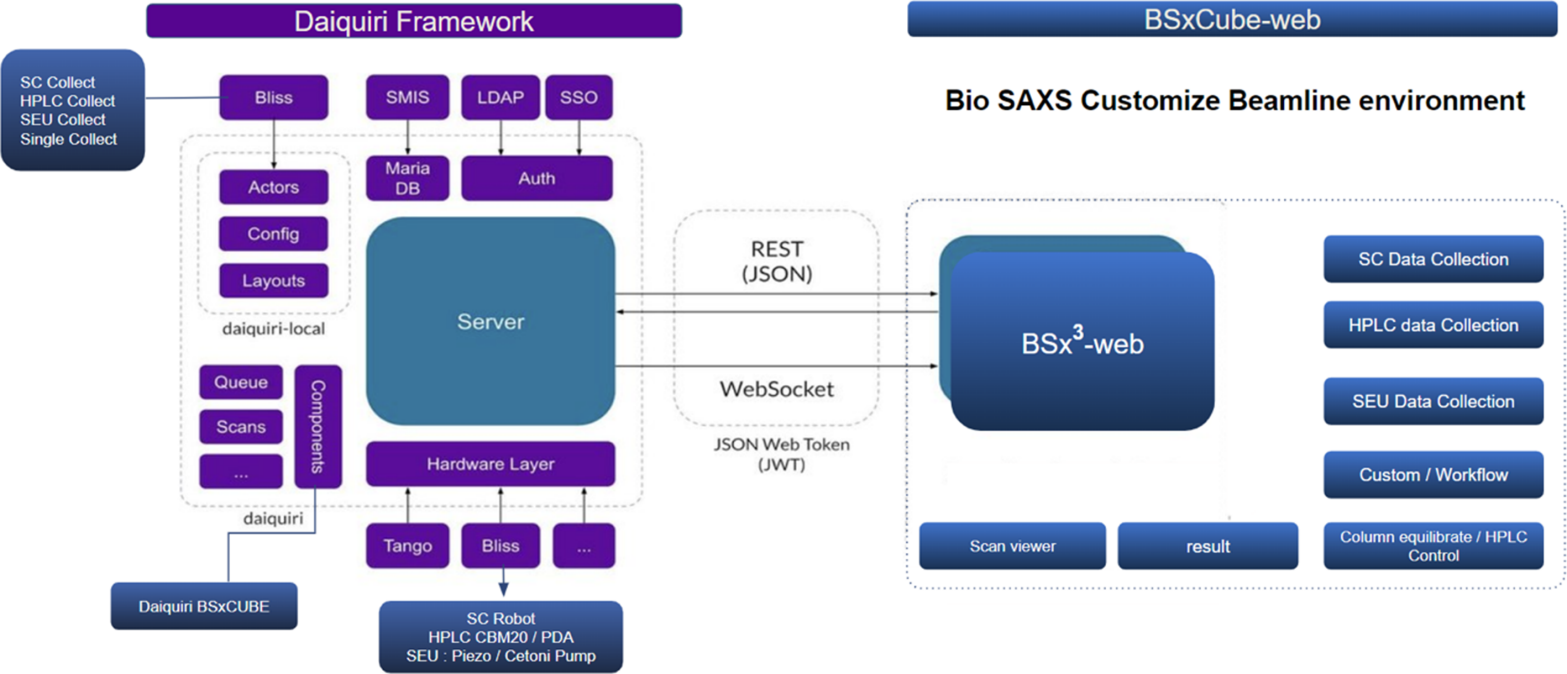
Schematic representation of the *Daiquiri* framework within the *BSxCuBE-Web* architecture.

The *BSxCuBE-Web* back-end is built on the *Daiquiri* framework (Fisher *et al.*, 2021 ▸), which provides a robust foundation for managing beamline hardware control, experimental data acquisition, and communication between multiple beamline components (Fig. 2 ▸). Within the *BSxCuBE-Web* architecture, *Daiquiri* functions as the middleware layer, enabling seamless communication between the web-based interface and the *BLISS* control software. Communication between *BSxCuBE-Web* and *Daiquiri* occurs through *RESTful* APIs using JavaScript Object Notation (JSON) for structured data exchange, while *WebSockets* enable real-time, bidirectional communication. The extensible architecture of *Daiquiri* supports the addition of new features and experimental workflows, making it possible to implement components tailored to bioSAXS experiments on BM29. Furthermore, *BSxCuBE-Web* shares its back-end infrastructure with other ESRF beamline applications, thereby simplifying maintenance and reducing development overhead.

The *BSxCuBE-Web* backend integrates multiple essential components of the beamline through *BLISS*, enabling control over various hardware devices and management of experimental procedures. *BLISS* (Guijarro *et al.*, 2023 ▸) allows Python packages or scripts to be loaded as external modules, providing control over beamline devices such as the sample stage, detector, sample changer robot, HPLC and SEU. This integration enables core beamline operations such as sample environment regulation, on-line sample purification via HPLC, and automation of complex measurement sequences.

A key aspect of the technology stack is the use of Python as a core programming language for both back-end development and data analysis pipelines. Python, with its extensive scientific libraries and versatility for scientific computing and system integration, ensures seamless communication between *BSxCuBE-Web* and various beamline components. In addition, Python facilitates integration of hardware components, making it ideal for orchestrating complex beamline operations. It can also be used to script and automate various functions required during data collection.

While *BSxCuBE-Web* primarily operates through the *BLISS* control system, the incorporation of the *Daiquiri* framework enhances adaptability by allowing communication with alternative control systems such as *Tango*, *EPICS* or other equivalent control frameworks. Furthermore, the modular nature of *Daiquiri* makes *BSxCuBE-Web* a scalable solution for beamline automation, regardless of the underlying control infrastructure. This adaptability ensures straightforward deployment of *BSxCuBE-Web* on bioSAXS beamlines at other synchrotron facilities.

*BSxCuBE-Web* implements input validation in both the client and server. On the client side, validation combines HTML5 built-in form checks, *React* component constraints, and schema-based validation using React JSON Schema Form (RJSF) to prevent invalid ranges, missing fields, and incorrectly formatted inputs before submission. On the server side, the Flask API enforces request and response validation using *Marshmallow* together with marshmallow-jsonschema, ensuring that all exchanged data conform to predefined schemas. This layered approach helps catch erroneous inputs early, reducing the risk of runtime failures during experiment execution, and improving overall reliability.

Overall, the combination of a *React*-based front-end, *Redux* state management, Python for backend control and data processing, and the powerful backend infrastructure based on *Daiquiri* and *BLISS* provides a reliable and powerful software solution for bioSAXS data collection and control at BM29. Crucially, this modular design directly translates into real-world benefits. By isolating complex hardware control from the user interface, it prevents UI interruptions from affecting ongoing data collection, maximizing reliability. This separation of concerns also significantly eases software maintenance and collaborative development. Furthermore, because the *Daiquiri* middleware abstracts the underlying hardware communication, the architecture is inherently adaptable, allowing the interface to be deployed at other synchrotron facilities by connecting to their local control systems. The combined use of modern web technologies and synchrotron-specific software frameworks enables *BSxCuBE-Web* to meet the demanding requirements of a modern bioSAXS beamline by facilitating high-throughput data collection with minimal user intervention.

## 5. Graphical user interface

**Figure 3:**
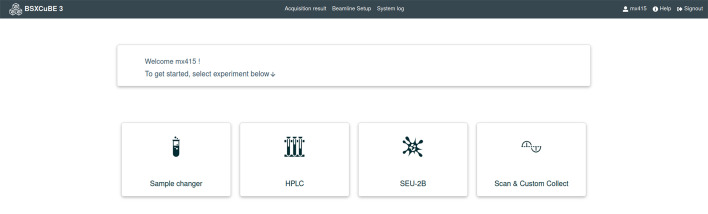
Home screen of *BSXCuBE-Web* showing the primary buttons for each experiment type available on BM29.

**Figure 4:**
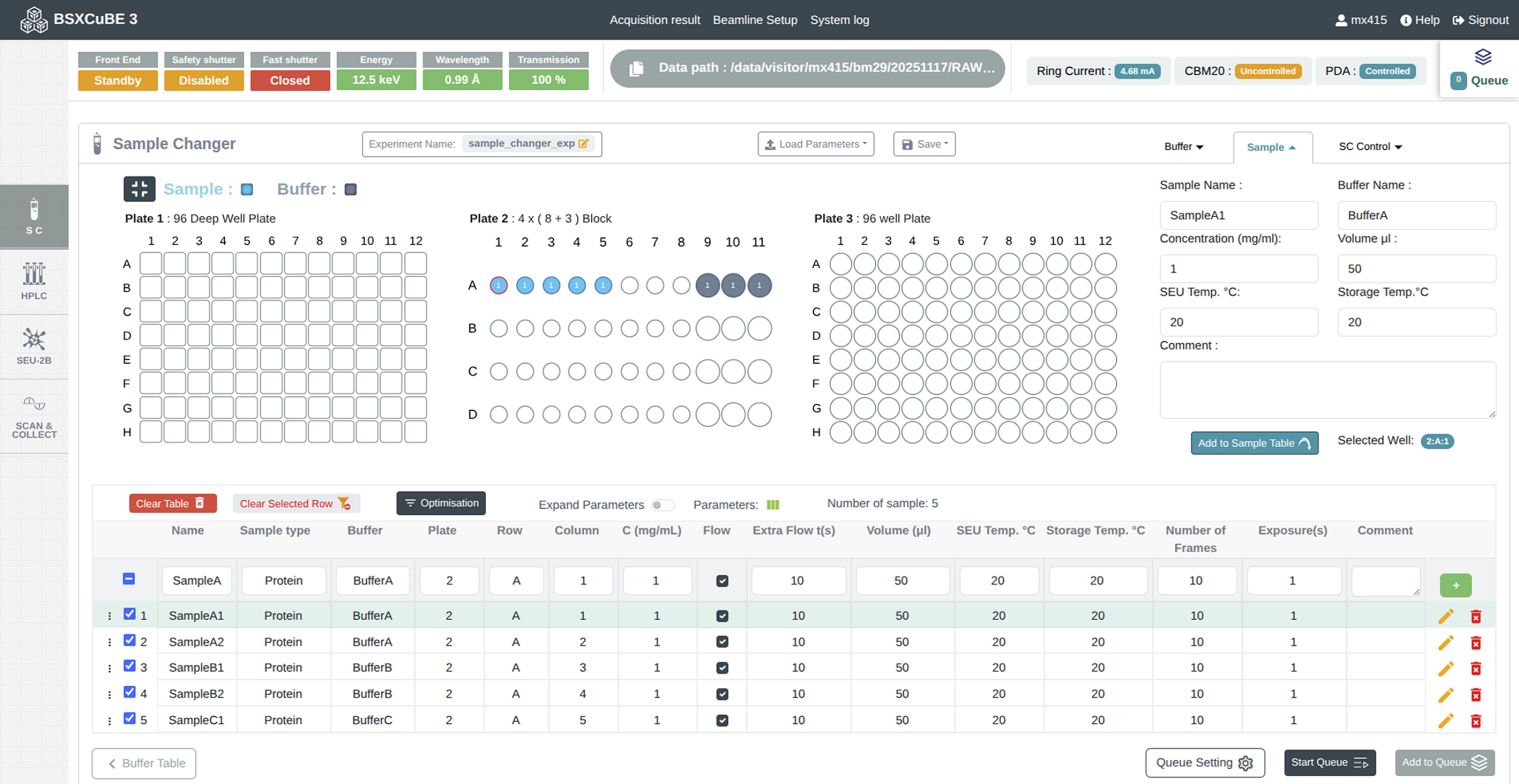
*BSxCuBE-Web* GUI for batch data collection using the bioSAXS sample changer. The layout mirrors the sample positioning in the sample changer robot to support intuitive configuration and automated queuing.

Adhering to the principle of a user-friendly and intuitive interface, the *BSxCuBE-Web* home screen prominently presents the experiment control for each beamline sample environment configuration (Fig. 3 ▸). The ‘Scan & Custom Collect’ button is primarily intended for staff and links to pages for beamline configuration and calibration. Once an experiment type is selected, a new tab opens with a header containing relevant beamline information, such as storage ring beam current and X-ray energy, which are coloured-coded in red, yellow, or green to indicate the beamline status (Fig. 4 ▸). The experiment type buttons remain present as a side bar on every experimental page, allowing users to switch between experiment types and monitor the beamline status.

### 5.1. Data collection tabs and experimental queue

The data collection tabs provide access to high-throughput workflows, making it possible to collect data from hundreds of samples within a single experimental session using an automated experimental queuing mechanism. By integrating different sample environments and robotic systems, such as the bioSAXS sample changer and HPLC system available on BM29,*BSXCuBE-Web* manages large-scale sample measurements efficiently while maintaining systematic consistency. The queuing system is a core component of *BSxCuBE-Web* to enable fully unattended, high-throughput data collection. It allows users to prepare, monitor, and manage a sequence of experiments efficiently; specifically, measurements from each sample environment can be queued from the SC and SEC-SAXS, along with equilibration steps and single measurements when required. The SEU-2B can also be run through a queue, but must be scheduled independently because it requires a distinct experimental setting.

#### 5.1.1. Data collection using the ESRF–EMBL bioSAXS sample changer

For high-throughput and efficient sample collection, BM29 utilizes the EMBL-designed bioSAXS sample changer, developed in collaboration with the ESRF (Round *et al.*, 2015 ▸) and commercially available from ARINAX (Moirans, France). This bioSAXS sample changer is specifically engineered to handle a large number of biological solution samples with minimal user intervention, facilitating rapid and reliable data acquisition for bioSAXS experiments. The robot operates via a Java based proprietary control GUI software (Round *et al.*, 2015 ▸) and includes motor controllers communicating via *TwinCAT* protocols, all running on a dedicated Windows-based PC. *BSxCuBE-Web* provides precise control over automated sample handling by directly controlling the bioSAXS robot, reducing the risk of contamination and ensuring repeatability of measurements. In addition, it replicates the layout of the three sample holders within the robot, enabling intuitive operation (Fig. 4 ▸).

The robotic sample changer can handle a wide variety of sample types, including proteins, nucleic acids, lipids, and other biological macromolecules in aqueous solutions. Under basic bioSAXS operation, each sample must be paired with a corresponding buffer position for background subtraction. This is easily achieved by switching between the ‘Buffer’ and ‘Sample’ tabs and selecting the appropriate position for each sample/buffer in the sample holder, followed by assigning the buffer through a handy drop-down menu. This pairing can then be added to the sample table, which supports different measurement configurations per sample type. Each entry in the sample table can be edited or deleted if required. Once the sample table is configured, it can be added to the ‘Queue’ and executed through the ‘Run’ command to start an automated data collection.

#### 5.1.2. Data collection using HPLC (SEC-SAXS)

**Figure 5:**
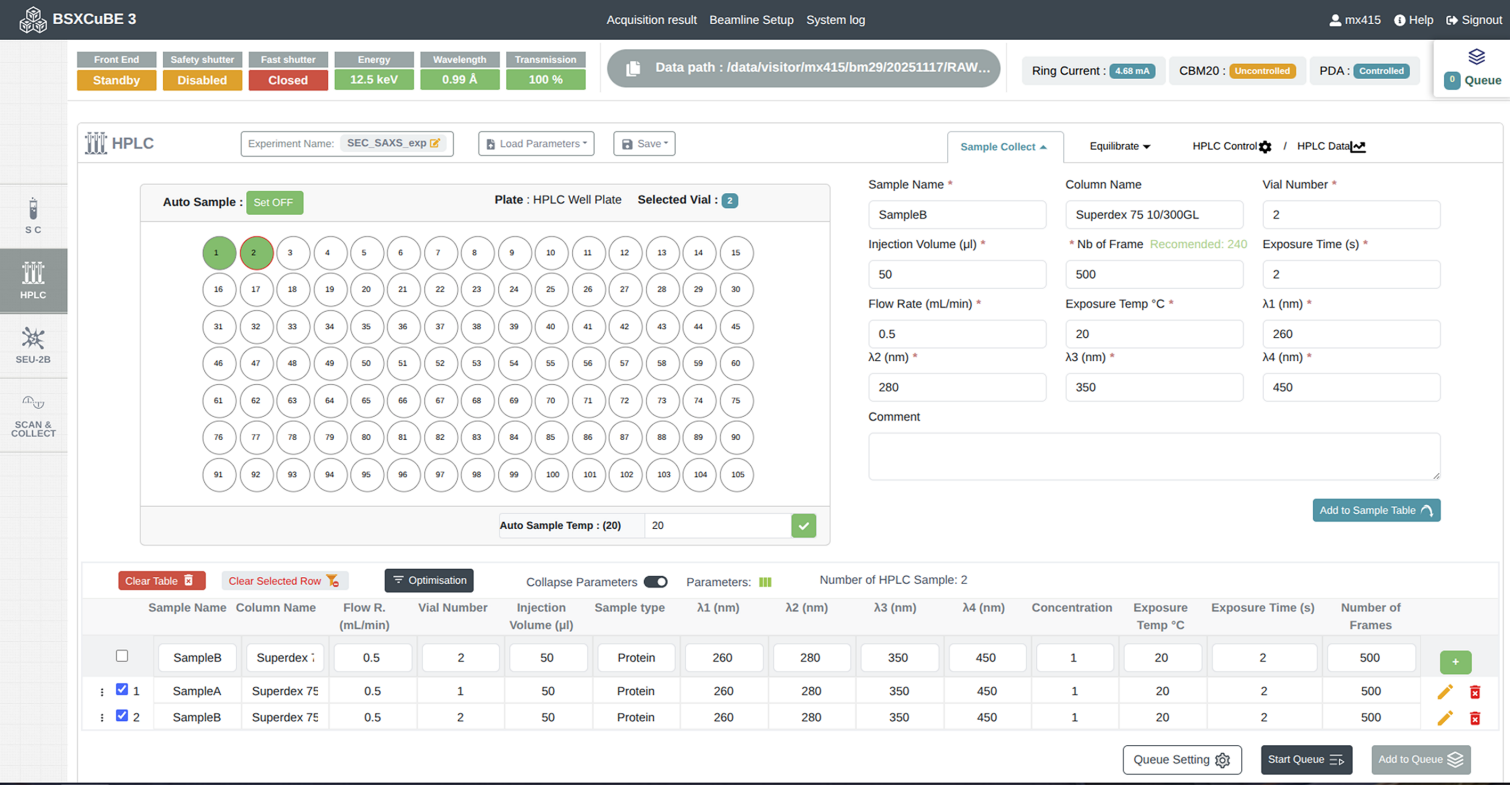
SEC-SAXS interface within the *BSxCuBE-Web* GUI showing sample holder and data entry tabs for Sample Collect, Equilibration, and HPLC Control panels, with real-time system diagnostics.

SEC is routinely employed to enable online purification and analysis of biological macromolecules prior to SAXS measurements, ensuring that only homogeneous, high-quality samples are analysed. SEC-SAXS is particularly beneficial for proteins and other macromolecules that may degrade or aggregate (Pérez *et al.*, 2022 ▸). To facilitate SEC-SAXS experimental measurements, an HPLC system (Shimadzu Corp., Kyoto, Japan) is integrated into *BSxCuBE-Web* within a dedicated sample environment tab, which includes controls for sample position selection, column equilibration, buffer management and HPLC operation (Fig. 5 ▸). This tab provides full access to HPLC functions controlled through the *BSxCuBE-Web* interface, including pump flow, pump wash, pressure readout, temperature regulation, as well as automated sample loading and injection onto the SEC column. A real-time plot within the interface displays column flow rate and current pressure in the system, allowing users to monitor chromatographic stability during data collection.

#### 5.1.3. Experimental queue implementation in *BSxCuBE-Web*

**Figure 6:**
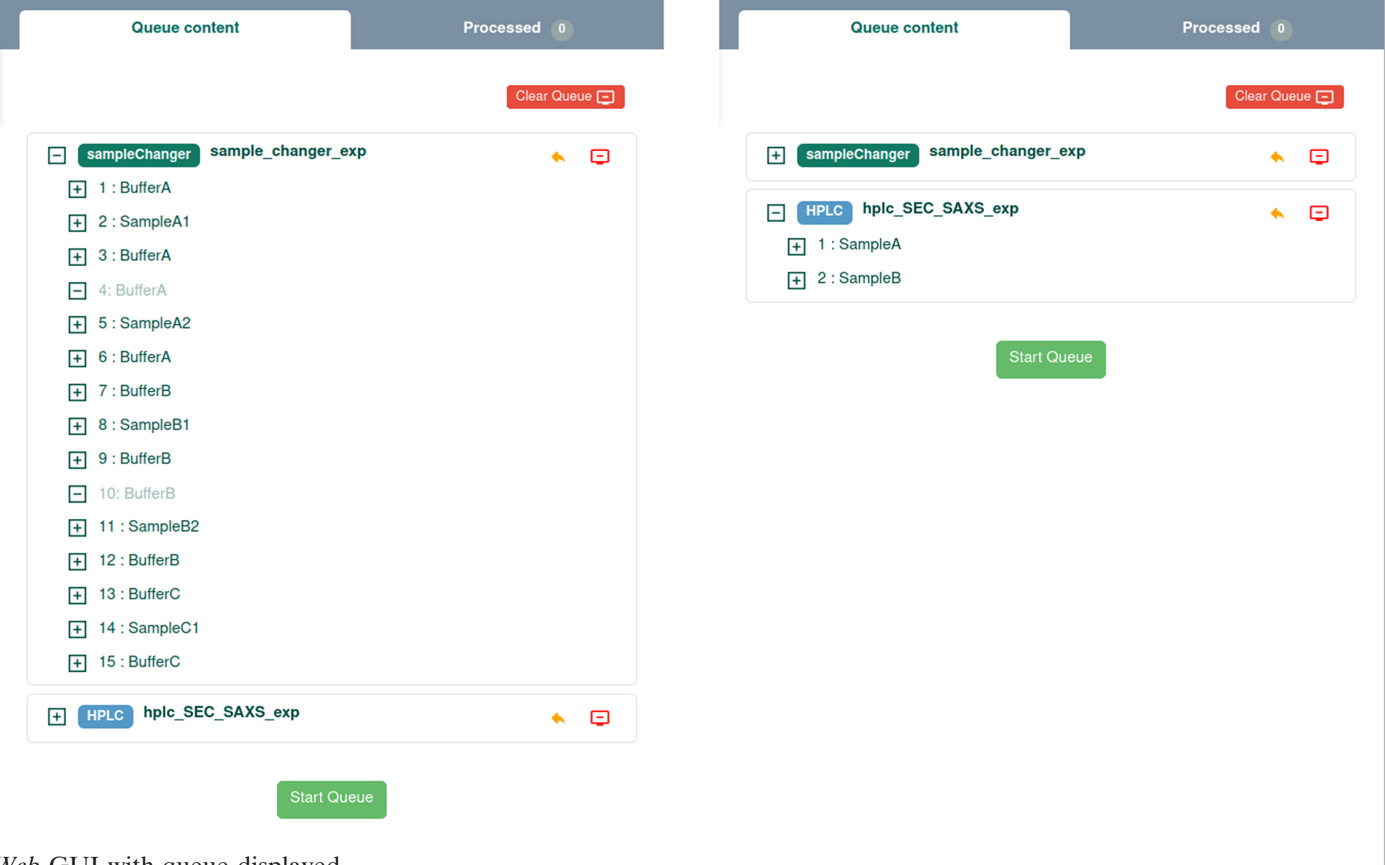
*BSxCuBE-Web* GUI with queue displayed.

A dedicated queue button, located in the upper-right corner of the GUI, becomes active when a data collection is added or explicitly opened by the user (Fig. 6 ▸). The queue panel provides a clear overview of all scheduled experiments, displaying their order, execution status (*e.g.* pending, running, completed, failed), and key metadata such as sample ID and data collection parameters. Users can edit, reorder, or remove any queued item prior to execution, enabling rapid adaptation to changing experimental priorities. The queue system is designed for robust operation, maintaining a persistent execution log and providing manual controls to abort individual data collections or terminate the entire queue process. For convenience, users may also import batch definitions directly from sample description files (formatted as either JSON or CSV) to populate the queue. This offline preparation is especially advantageous for time-critical sessions, complex experimental designs, and remote mail-in experiments, as it allows users to fully define their data collection parameters in advance of their beam time. A key feature is graceful error recovery. Depending on the nature of the error, different recovery strategies are applied. Recoverable, sample-specific errors may result in skipping to the next experiment, allowing unattended data collection to continue. In contrast, hardware faults always trigger a full stop of the queue to ensure experimental safety. Transient events such as a temporary beam loss trigger a pause, allowing the experiment to resume once stable beam conditions are restored. These queue errors are automatically logged, providing the beamline staff and users with detailed diagnostic feedback. Together, all these features make the *BSxCuBE-Web* queue a powerful tool for maximizing beam-time productivity and minimizing manual intervention.

#### 5.1.4. Data collection using the sample exposure unit 2B

The recently integrated SEU-2B further expands the capabilities of BM29, enabling more complex bioSAXS experiments that require controlled positioning and alternative sample formats (Tully *et al.*, 2023 ▸; Blanchet *et al.*, 2025 ▸). The SEU-2B supports a more specialized sample environment, including different types of customized sample holders such as microfluidic devices, and it is designed for experiments that require precise exposure conditions. One of the key features of the SEU-2B is the ability to perform 2D scans using piezo motor stages to target specific areas of interest within a mounted sample. This level of spatial precision, enabled by the integration of the piezo motor control in *BLISS*, allows for the analysis of complex sample types such as biological gels or liquid–liquid phase separation (LLPS) condensates. Furthermore, the integration of CETONI Nemesys P (CETONI GmbH, Korbussen, Germany) pump control via *BLISS* also allows their precise control for studying dynamic biological processes. Together, these developments will allow the collection of time-resolved bioSAXS data from complex biological systems within a confined microfluidic device.

**Figure 7:**
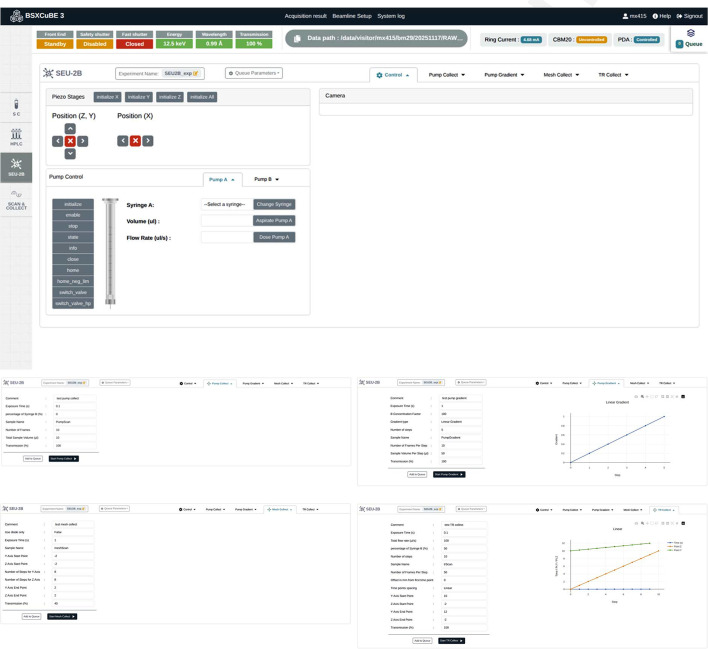
The SEU-2B tab in the *BSxCuBE-Web* GUI, enabling 2D scan definition and microfluidic coordination for advanced bioSAXS workflows. Different tabs allow control of different experiment types including pump collect, gradient collect, mesh collect, and time-resolved collect.

*BSxCuBE-Web* has been further developed to allow users to define and control SEU-2B scans, specifying the exact points or regions to be scanned and optimizing exposure parameters for each location. This is highly advantageous for researchers who need to examine fine-scale heterogeneities or study reaction kinetics under controlled flow conditions within microfluidic devices. Importantly, these advanced capabilities in *BSxCuBE-Web* are made accessible through the same intuitive interface, lowering the barrier of use for non-experts (Fig. 7 ▸). This combination of advanced sample environment control and user-friendly automation significantly broadens the scope of BM29, facilitating novel studies in structural biology and material sciences where traditional static sample environments would be insufficient.

#### 5.1.5. Single collect/scan and collect

**Figure 8:**
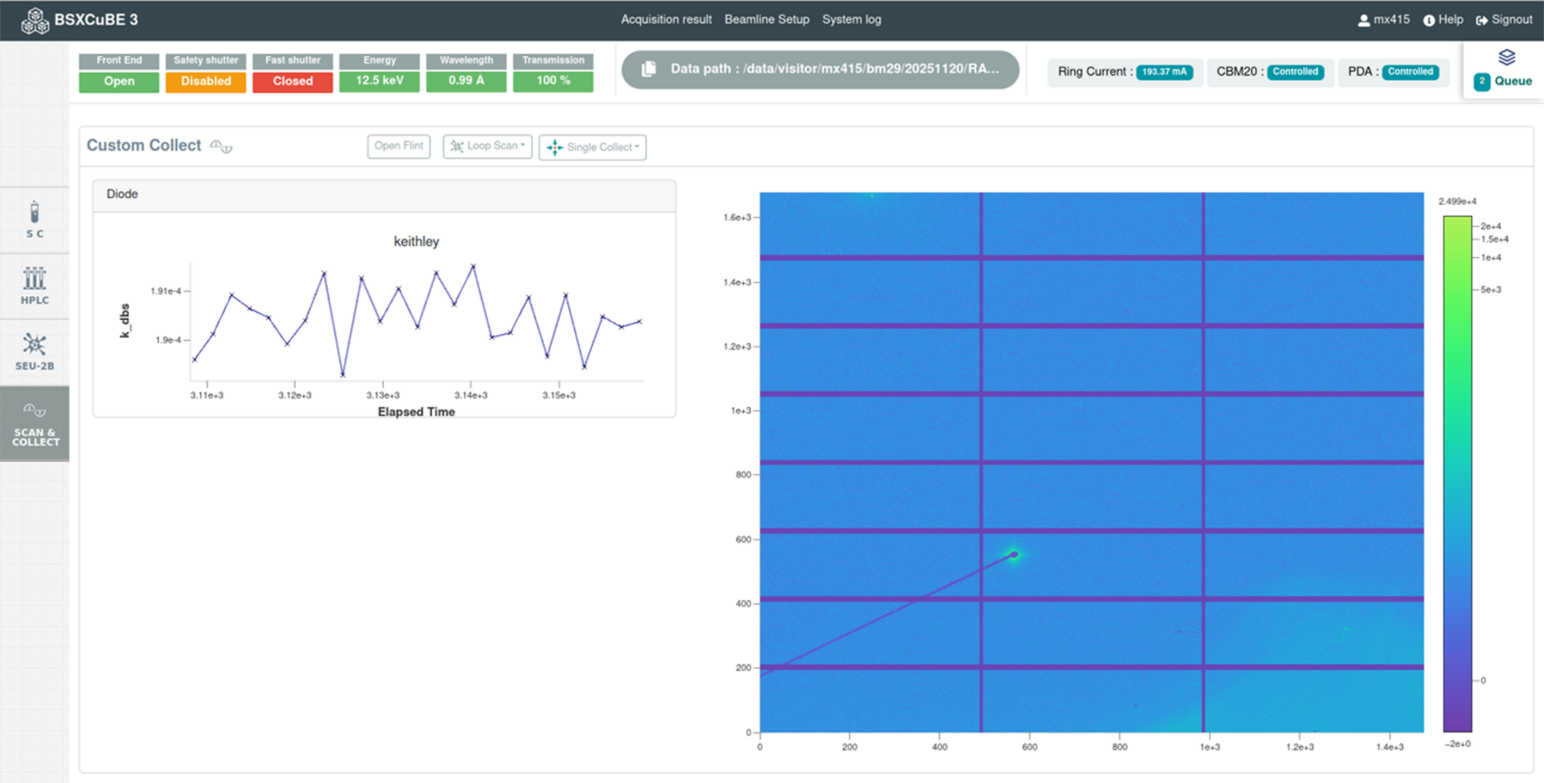
The *BSxCuBE-Web* GUI provides a real-time scan visualization using h5web integration.

For smaller-scale experiments or more specialized cases, users can manually inject samples directly into the capillary using either the bioSAXS sample changer or manually with a syringe. This option offers flexibility for unique or highly sensitive samples that may not be compatible with automated workflows or SEC-SAXS setups. These manual data collection modes have been implemented in *BSxCuBE-Web* to ensure support for a broad range of experimental workflows, including highly customized manual setups that accommodate diverse user needs (Fig. 8 ▸).

To streamline such data collection modes, *BSxCuBE-Web* features real-time scan visualization and diode signal plotting, leveraging the h5web library (Bocciarelli *et al.*, 2022 ▸) for interactive and dynamic data representation (Fig. 8 ▸). While this implementation provides immediate feedback during experiments, ongoing developments aim to enhance responsiveness and interactivity. Further upgrades will focus on refining the user interface and expanding visualization to better support advanced SAXS measurement workflows.

### 5.2. Data analysis pipelines

*BSxCuBE-Web* uses an efficient and automated data analysis pipeline to process SAXS data in real time, allowing users to quickly assess the quality of their results. The pipeline relies on *Dahu*, an open-source software framework that uses efficient algorithms implemented in *FreeSAS* (Kieffer *et al.*, 2022 ▸), which provides a comprehensive set of tools for SAXS data processing and analysis. The connection between *BSxCuBE-Web* and *Dahu* is managed by *EWOKS*, a workflow execution engine designed at ESRF to build and run modular data processing workflows (De Nolf *et al.*, 2024 ▸). *EWOKS* wrappers ensure that the different steps of the SAXS pipeline are executed reliably and in the correct order, while also providing flexibility for adding or customizing analysis tasks.

After data collection, raw scattering curves undergo automatic processing, including background subtraction, data averaging, and normalization. Advanced data analysis tools, such as *Guinier* fitting, molecular weight estimation, and pair distribution function [*P*(*r*)] calculations, are then applied to extract the structural characteristics of the sample. The combination of *EWOKS* workflow orchestration and the robust, fast algorithms of *FreeSAS* ensures that accurate and reproducible SAXS analysis results are obtained quickly. This reduces manual effort and accelerates the path from data collection to meaningful data interpretation, improving overall experimental efficiency.

Essential parts of the processed data can be visualized directly through the *BSxCuBE-web* interface, giving immediate feedback to users (Figs. 8 ▸ and 9 ▸). A more detailed inspection of current and previous data collections is available using ISPYB-Exi for bioSAXS (De Maria Antolinos *et al.*, 2015 ▸) and via the new ESRF ISPyB-DRAC experimental database (De Maria *et al.*, 2024 ▸). These two databases also allow users to download their datasets.

## 6. Conclusion and future perspectives

The development and deployment of *BSxCuBE-Web* represents the culmination of nearly seven years of continual development and optimization. While the *MxCuBE* international consortium (Oscarsson *et al.*, 2019 ▸) provided an initial template, its adaption to bioSAXS beamline control required the resolution of significant technical challenges, spanning from computing infrastructure to application performance. A foundational hurdle was the outdated beamline control environment, which relied on a deprecated operating system (*Debian 8*) and an obsolete web browser. This legacy setup lacked compatibility with modern web standards and posed significant security risks. This was addressed by spearheading a necessary infrastructure migration to the latest version of *Ubuntu* and web browser, which provided a stable, secure, and performant foundation for the web-based application. Further upgrades on the infrastructure are foreseen to continue to maintain an optimal user experience and support the evolving needs of high-throughput bioSAXS experiments.

On the application side, a major challenge was efficient browser-side memory management. The continuous rendering of large, real-time data streams in *Chrome* led to progressive memory leakage and reduced interface responsiveness over prolonged sessions. This issue was mitigated by optimizing the rendering pipeline through lazy-loading components and by adopting the high-performance plotting library, h5web. These changes significantly improved stability and responsiveness during long-running experiments. Synchronization between the GUI, *Daiquiri*, and *BLISS* also posed initial challenges. Early versions exhibited latency in status updates, creating inconsistencies between the GUI and the actual beamline state. We resolved this by refining the *WebSockets* protocol for near real-time push updates and implementing the redundant *REST* API for critical state polling as a fallback synchronization mechanism.

Retrospective analysis identified opportunities for considering alternative state management approaches as the project evolves. While *Redux* and *WebSockets* are a reliable and effective foundation for data synchronization, newer reactive solutions (*e.g.TanStack Query* or *RxJS*) offer additional features that could further simplify asynchronous data handling and reduce the amount of custom logic required. These options may therefore be worth evaluating in future iterations of *BSxCuBE-Web* to continue improving scalability and maintainability. Integrating automated performance profiling earlier in development would likewise have accelerated the identification and resolution of browser-specific bottlenecks. Importantly, addressing these challenges has ultimately enhanced the application’s robustness and provided invaluable lessons for future beamline GUI development, emphasizing the need for modern infrastructure, proactive performance optimization, and resilient communication patterns in user experimental measurements at synchrotrons.

In conclusion, *BSxCuBE-Web* has substantially enhanced experimental efficiency and user accessibility at the ESRF BM29 beamline by providing an intuitive, web-based platform for automated and simplified bioSAXS data collection. Its integration of automation, real-time feedback, modular experimental control and compatibility with various control frameworks, such as *BLISS* and *EPICS*, supports both routine and advanced workflows for expert and non-expert users alike.

Looking forward, several enhancements and further automation are anticipated on BM29; notably, the integration of new sample exposure setups, including microfluidic devices, some of which have already been demonstrated but that still require extensive testing to ensure robust functionality. These new experimental capabilities will likely require the addition of new experimental control features to *BSxCuBE-Web*, such as temperature-controlled cells for more adaptive and advanced technologies. Furthermore, improvements to visualization tools (*e.g.* Scan Viewer, Result Display) and long-term application resilience are also foreseen, ensuring *BSxCuBE-Web* will remain performant with a reliable, efficient, and state-of-the-art data collection platform.

Another key area for future development in *BSxCuBE-Web* is the enhancement of metadata acquisition during bioSAXS experiments. By expanding the scope and precision of the metadata collected, such as experimental parameters, sample characteristics, beamline settings, and environmental conditions, *BSxCuBE-Web* will support comprehensive and well structured datasets. An enriched metadata will not only improve the traceability and reproducibility of experiments but will also serve as a foundation for implementing machine learning techniques in data processing pipelines, aimed at automated complex data processing tasks, optimized experimental workflows, and extraction of patterns or insights that would otherwise require extensive manual analysis.

Continued collaboration between ESRF and EMBL Grenoble will ensure that *BSxCuBE-Web* remains at the forefront of bioSAXS beamline control and data acquisition and will pave the way for more advanced, efficient, and automated bioSAXS experiments at synchrotron facilities.

## Figures and Tables

**Figure 9:**
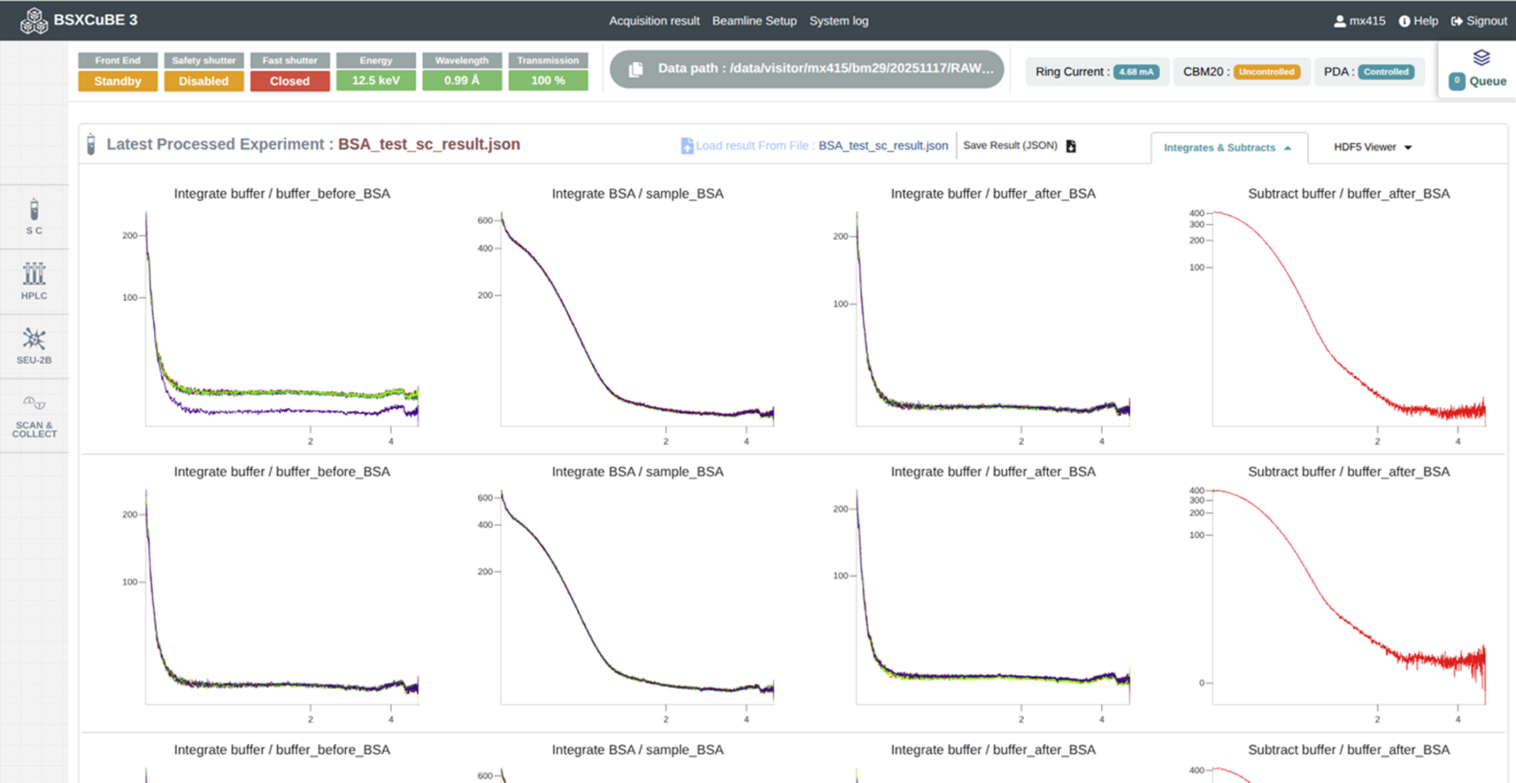
*BSxCuBE-Web* GUI representation of data processing results from *FreeSAS* (Kieffer *et al.*, 2022 ▸).

**Table 1:** List of bioSAXS user control software used at synchrotron radiation facilities

Facility / beamline	Primary control software	Core technology / language	User interface paradigm	References
ALBA, Spain / BL11-NCD-SWEET	*Spock*	*IPython*	Command line interface	BL11-NCD-SWEET link
Advanced Light Source, USA / SIBYLS	SEC-SAXS: specialized UI HT-SAXS: *Blu-Ice for SAXS*	*DCS*, Tcl/Tk, C++	Desktop client	(Rosenberg *et al.*, 2022 ▸; Classen *et al.*, 2010 ▸)
Advanced Photon Source, USA / BioCAT	Specialized UI		Desktop client	BioCAT beamline link
Australian Synchrotron, Australia / BioSAXS	*Bluesky*	Python	Desktop client	(Allan *et al.*, 2019 ▸)
Cornell High Energy Synchrotron Source (CHESS), USA / XBio	Command line / Robocon UI	*EPICS, SPEC, Python*	Command line interface	(Acerbo *et al.*, 2015 ▸)
Diamond Light Source, UK / B21	*Generic Data Acquisition* (*GDA*)	*EPICS*, Java, *Eclipse*, Python	Desktop client	(Cowieson *et al.*, 2020 ▸)
EMBL-Hamburg @ PETRA III, Germany / P12	*BECQUEREL*	C++, *Qt*	Desktop client	(Hajizadeh *et al.*, 2018 ▸)
European Synchrotron Radiation Facility (ESRF), France / BM29	*BSxCuBE* (pre 2019), * BSxCuBE-Web* (since 2019)	Python, *Qt3 Python*, *BLISS*	Desktop client web browser	(Pernot *et al.*, 2013 ▸), this work
National Synchrotron Light Source-II, USA / LiX	*Bluesky*	Python	Desktop client	(Allan *et al.*, 2019 ▸)
National Synchrotron Radiation Research Center (NSRRC), Taiwan / TPS-13 A	*DC-GUI*	*EPICS*, *SPEC*	Desktop client	(Shih *et al.*, 2022 ▸)
Photon Factory, Japan / BL-10C and BL-15A2	Specialized UI under *STARS* framework	Python, Perl, Java	Desktop client	(Shimizu *et al.*, 2019 ▸)
Synchrotron Soleil, France / SWING	Specialized UI	*PasserelleIDE* (Java-based), *TANGO*	Desktop client	(Thureau *et al.*, 2021 ▸)
Shanghai Synchrotron Radiation Facility, China / BL19U2	*CSS-BOY* UI	*EPICS*, *CS-Studio*	Desktop client	(Li *et al.*, 2016 ▸)
Stanford Synchrotron Radiation Lightsource, USA / BL4-2	*Blu-Ice for SAXS*	Tcl/Tk, C++	Desktop client	(McPhillips *et al.*, 2002 ▸)

## Data Availability

BSxCuBE-Web is open-source, and its code is freely available at https://gitlab.esrf.fr/ui/bsx3.
